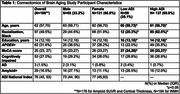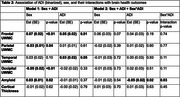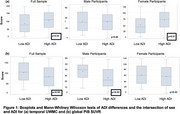# Intersections of sex and neighborhood disadvantage on Alzheimer's disease pathology

**DOI:** 10.1002/alz70860_104064

**Published:** 2025-12-23

**Authors:** Alexandria C. Reese, Sarah K. Royse, Beth E. Snitz, Nirupama Natarajan, James Hengenius, Rebecca E. Roush, Geraldine Cisneros, Alexandra Gogola, Brian J Lopresti, Oscar L Lopez, James T Becker, Ann D Cohen, C. Elizabeth Shaaban

**Affiliations:** ^1^ University of Pittsburgh, Pittsburgh, PA, USA; ^2^ University of Pittsburgh Alzheimer's Disease Research Center (ADRC), Pittsburgh, PA, USA; ^3^ CHU Sainte‐Justine Research Centre, Université de Montréal, Montréal, QC, Canada; ^4^ University of Pittsburgh, School of Medicine, Pittsburgh, PA, USA

## Abstract

**Background:**

Differences in Alzheimer's disease (AD) biomarkers have been reported by sex, but their intersection with neighborhood disadvantage is not understood.

**Method:**

Participants were enrolled in the Connectomics of Brain Aging study. Neighborhood disadvantage was assessed using the area deprivation index (ADI) national rank and was binarized via 50^th^ percentile, where higher scores indicate greater disadvantage. β‐amyloid (A) was measured via global ^11^C‐PiB SUVR. Our vascular (V) measure was lobar unhealthy white matter connectivity (UWMC), a novel measure indicating the proportion of white matter connections affected by white matter hyperintensities (WMH). Neurodegeneration (N) was measured via cortical thickness from an AD‐specific meta‐region. Intersectional effects of sex and ADI on AV(N) were examined on the multiplicative and additive scale via robust and log‐binomial regressions, respectively.

**Result:**

The sample included *N* = 196 participants (age 62 years; female sex 67%; Black racialization 49%; education 14 years; *APOE4+* 32%; cognitively impaired 27%; Table 1). In robust regressions, female participants had greater frontal UWMC (β=0.07, SE=0.02, *p* <0.01) and amyloid (β=0.03, SE=0.01, *p* = 0.02) but lower parietal (β=‐0.03, SE=0.01, *p* = 0.04) and occipital UWMC (β=‐0.05, SE=0.02, *p* <0.01) versus male participants. High ADI participants had greater frontal (β=0.05, SE=0.02, *p* = 0.01) and temporal UWMC (β=0.03, SE=0.02, *p* = 0.05) versus low ADI participants (Table 2). Intersectional effects were found such that high ADI female participants had the greatest temporal UWMC on the additive scale (relative excess risk due to interaction = 0.68, 95% CI=[0.12,1.25]), and low ADI men had the greatest amyloid on the multiplicative scale (*p*‐interaction = 0.03). Sex‐stratified regressions indicated that high ADI was associated with greater temporal UWMC in female participants (β=0.05, SE=0.02, *p* = 0.01), but not male participants (β=‐0.003, SE=0.03, *p* = 0.92; Figure 1a) and low ADI was associated with greater amyloid in male participants (β=‐0.06, SE=0.02, *p* = 0.02) but not female participants (β=0.01, SE=0.02, *p* = 0.74; Figure 1b). Results examining WMH instead of UWMC were similar.

**Conclusion:**

We confirmed differences in AV(N) by sex and ADI. Intersectional effects were found such that women with high ADI had the greatest temporal UWMC and men with low ADI had the greatest amyloid. Understanding the role of neighborhood disadvantage on V is important for future interventions, especially for women.